# Isolation of farnesylhydroquinones from the basidiomycete *Ganoderma pfeifferi*

**DOI:** 10.1007/s13659-013-0036-5

**Published:** 2013-06-20

**Authors:** Timo H. J. Niedermeyer, Thomas Jira, Michael Lalk, Ulrike Lindequist

**Affiliations:** 1 Institute of Pharmacy, Ernst-Moritz-Arndt-University, Friedrich-Ludwig-Jahn-Straße 17, 17487 Greifswald, Germany; 2 Present Address: Institute of Biochemistry, Ernst-Moritz-Arndt-University, Felix-Hausdorffstraße 4, 17487 Greifswald, Germany

**Keywords:** *Ganoderma pfeifferi*, farnesylhydroquinone, ganomycin

## Abstract

Two farnesylhydroquinones were isolated from the fruiting bodies of *Ganoderma pfeifferi*, farnesylhydroquinone (**1**) and the new compound ganomycin K (**2**), (5*S*)-3-[(*E*)-7,8-dihydroxy-4,8-dimethylnon-3-enyl]-5-(2,5-dihydroxyphenyl)-furan-2(5*H*)-one. The structures of **1** and **2** were determined on the basis of mass spectrometric and NMR spectroscopic evidence. The antibacterial activity of the isolated compounds was neglectable.

**Electronic Supplementary Material:**

Supplementary material is available for this article at 10.1007/s13659-013-0036-5 and is accessible for authorized users.

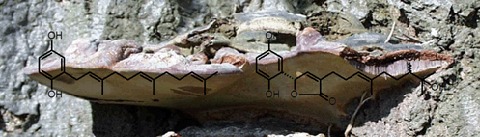

## Introduction

*Ganoderma pfeifferi* Bres., a weak parasitic and later saprophytic basidiomycete, is a fungus only found in Europe, living preferentially on *Fagus* spp. and some other deciduous trees such as *Aesculus*, *Acer*, *Fraxinus*, and *Quercus* species. The fungus can be recognized by its cracked and wrinkled resinous layer on the pileus and its sweet scent in winter. Its dark brown context makes it distinguishable from older specimens of *G. lucidum* and *G. resinaceum*.[1]

In contrast to *G. lucidum* and *G. applanatum*, from which a number of biologically and pharmacologically interesting triterpenes and polysaccharides have been isolated,[2,3] *G. pfeifferi* is one of the phytochemically less well examined species of the family Ganodermataceae. Two antibacterial farnesylhydroquinones were isolated from *G. pfeifferi*,[4] followed by the isolation of antiviral triterpenes.[5,6] In the course of the isolation of larger amounts of the ganomycins A and B from *G. pfeifferi* for subsequent bioactivity studies, several other compounds with comparable UV/Vis spectra have been observed in chromatograms of various extract fractions. Of these compounds, two were characterized in more detail. In addition to the known compound farnesylhydroquinone (**1**), we characterized a previously unknown compound we named ganomycin K (**2**). The present manuscript describes the isolation and structure elucidation of these compounds as well as the biological examination of the compounds in regard to their antibacterial activity.

*To whom correspondence should be addressed. E-mail: timo.niedermeyer@uni-greifswald.de

**1**

**2**

## Results and Discussion

The first compound (**1**) eluted later than the known ganomycins A and B on a $C_{18}$ HPLC column ($t_r$ **1**: 9.2 min, $t_r$ ganomycin A: 7.2 min, $t_r$ ganomycin B: 8.5 min). It was obtained as a brown oil. GC-EIMS of **1** showed a molecular ion peak $[M]^+$ at *m/z* 314. The $^1$H NMR spectrum of **1** showed close similarities with the spectrum of ganomycin B and revealed the presence of the hydroquinone as well as the double bonds in the terpene part of the molecule. However, the low-field resonance of the methine proton conjugated to the carboxylic acid in ganomycin B was missing, but instead a methine proton at higher field closer to the other methine proton was observed. Furthermore, instead of three methyl signals in ganomycin B, four methyl group singulets were observed for **1**. Thus, **1** has been identified as farnesylhydroquinone. Its NMR data are comparable with data published in the literature.[7,8] To confirm the identity of **1**, farnesylhydroquinone has been synthesized. The synthetic compound showed the same GC retention time and EI mass spectrum. Farnesylhydroquinone (**1**) has previously been isolated from marine organisms such as algae[9] and higher plants from the families Hydrophyllaceae[7] and Piperaceae[8], and similar compounds have been obtained from sponges[10] and other plants[11]. Although a related natural product, 2-methyl-farnesylhydroquinone, has been found in *Penicillium* sp.,[12] to the best of our knowledge this is the first report of farnesylhydroquinone being isolated from a fungus. However, the occurrence of farnesylhydroquinone in *Ganoderma pfeifferi* is not very surprising, as it can be supposed to be the biosynthetic precursor of the ganomycins.

Compound **2** eluted earlier than the known ganomycins on a $C_{18}$ HPLC column ($t_r$ **2**: 6.4 min) and was obtained as a yellow oil. The compound was not stable under the GC-EIMS conditions used. From LC-IT-TOFMS, a molecular formula of $C_{21}H_{28}O_6$ was established for **2** (found *m/z* 375.1820, calcd 375.1813 for $[M - H]^-$, Δ 0.7 ppm). The $^1$H NMR spectrum of **2** showed some similarities with the spectrum of the ganomycins, e.g. the presence of the hydroquinone as well as methyl groups, but it also showed marked differences (NMR data see Table 1). Three methyl group protons were observable. In contrast to the ganomycins A and B, only one methine proton resonance was observable in the range of 5.0 to 6.0 ppm, but two additional signals were observed at $\delta$ 7.35 ppm (H-2) and $\delta$ 6.23 ppm (H-1), according to the COSY spectrum corresponding to directly adjacent protons. The low field resonances indicated strong electron drawing mesomeric and/or inductive influences on these protons. Based on COSY (no additional protons in this spin system), HSQC (H-1 is attached to an alcoholic carbon at $\delta$ 80.0 ppm, H-2 is attached to a carbon at $\delta$ 151.2 ppm, indicating a double bond under mesomeric influence of a carbonyl group) and HMBC data (correlations between H-1 and a hydrogen-free $sp^2$ carbon at $\delta$ 133.3 ppm and between H-2 and a carbonyl carbon at $\delta$ 177.0 ppm as well as a $sp^3$ carbon triplet at $\delta$ 27.1 ppm), these non-aromatic protons could be assigned to an unsaturated isoprene lactone, and HMBC correlations from the proton H-4 extended into yet another attached unmodified isoprene unit. Indeed, the proton and carbon shifts of the postulated hydroquinone-isoprenelactone fragment in **2** agree very well with the NMR data of the corresponding fragment in ganomycin I, previously described from *Ganoderma colossum*.[13] However, two additional carbon signals at $\delta$ 74.0 ppm and 79.2 ppm, according to HMBC correlations belonging to two vicinal carbons, indicated the presence of two carbon-oxygen bonds not present in ganomycin I. These observed shifts correspond well to those of a dihydroxy isoprene moiety observed in other natural products.[14–16] The identified hydroquinone-isoprenelactone and dihydroxy isoprene moieties could be connected based on several HMBC correlations as indicated in Table 1 to give the planar structure of **2**. The absolute configuration at C-1 was established using the circular dichroic method described for the determination of the absolute configuration of 5-substituted 2-(5*H*)-furanones, where it is known that the sign of the Cotton effect due to the n-π* and π-π* transitions is directly correlated with the absolute configuration of the stereogenic center at the *γ*-carbon of the butenolide moiety.[17,18] The CD spectrum of **2** displayed a negative Cotton effect at 213 nm and a positive Cotton effect at 233 nm, indicating "M helicity" for the present lactone.[17] Therefore, the absolute configuration at C-1 could be assigned as *R*. The same configuration at C-1 has also been observed for ganomycin I.[13] Stereochemistry at C-10 could not be determined.

Taking into account all analytical data, compound **2** could

**Table 1. $^1$H and $^{13}$C NMR data for ganomycin K (2) in methanol-$d_4$ (600 MHz $^1$H NMR frequency)**

| position | $\delta_C$ | $\delta_H$ (*J* in Hz) | HMBC[a] | NOESY |
|---|---|---|---|---|
| 1' | 149.1, C | | | |
| 2' | 123.7, C | | | |
| 3' | 113.5, CH | 6.46, d, (2.8) | 1', 5', 1 | |
| 4' | 151.6, C | | | |
| 5' | 117.42, CH | 6.61, dd, (8.7, 2.9) | 1', 3' | 6' |
| 6' | 117.38, CH | 6.68, d, (8.6) | 2', 4', 1 | 5' |
| 1 | 80.0, CH | 6.23, s | 1', 2', 3', 2, 3 | 2 |
| 2 | 151.2, CH | 7.35, s | 1, 3, 4, 15 | 1, 4, 5 |
| 3 | 133.3, C | | | |
| 4 | 27.1, $CH_2$ | 2.35, m | 2, 3, 5, 6, 15 | 2, 6 |
| 5 | 26.3, $CH_2$ | 2.31, m | 3, 6, 7 | 2, 6, 8, 14 |
| 6 | 124.3, CH | 5.20, br. s | 4, 5, 8, 14 | 4, 5, 8 |
| 7 | 138.0, C | | | |
| 8 | 38.0, $CH_2$ | 2.00, m | 6, 7, 9, 10, 14 | 5, 6, 9, 10, 14 |
| 9 | 30.9, $CH_2$ | 1.31, m | 7, 8, 10, 11, 12 | 8, 10, 13, 14 |
| 10 | 79.2, CH | 3.22, ddd, (10.6, 3.5, 1.7) | 8, 9, 11, 12, 13 | 8, 9, 12, 13, 14 |
| 11 | 74.0, C | | | |
| 12 | 25.1, $CH_3$ | 1.11, s | 9, 10, 13 | 9, 10, 14 |
| 13 | 25.8, $CH_3$ | 1.14, d, (3.4) | 9, 10, 12 | 9, 10, 14 |
| 14 | 16.4, $CH_3$ | 1.60, s | 6, 7, 8 | 5, 8, 9, 10, 12, 13 |
| 15 | 177.0, C | | | |

[a]HMBC correlations are stated from proton to the indicated carbon.

be identified as (5*S*)-3-[(*E*)-7,8-dihydroxy-4,8-dimethylnon-3-enyl]-5-(2,5-dihydroxyphenyl)-furan-2(5*H*)-one. It was named ganomycin K.

In contrast to the positive control ampicillin and to ganomycin B, **1** did not show any antibacterial activity against the test strains at 100 $\mu$g/disc in the agar diffusion assay; at the same concentration, compound **2** exhibited moderate activity only against *St. aureus*, North German Epidemic Strain, and was inactive against the other strains tested. The assay results indicate that the free carboxylic acid group present in the ganomycins A and B seems to be important for antibacterial activity.

## Experimental Section

**General Experimental Procedures.** NMR spectra were recorded in $CDCl_3$ at 500 MHz ($^1$H NMR frequency; **1**) and methanol-$d_4$ at 600 MHz ($^1$H NMR frequency; **2**) at ambient temperature using a Bruker Avance-II 600. Spectra were referenced indirectly to tetramethylsilane via the residual signals of the deuterated solvent. ITTOF MS data have been acquired using an HPLC coupled to an ITTOF mass spectrometer (Shimadzu Europe GmbH, Duisburg, Germany) with electrospray ionization in negative mode, and were evaluated using the vendor's software LCMSSolution version 3.60.361 with Formula Predictor version 1.13. Precursor ions corresponding to $[M - H]^-$ were isolated in the ion trap and separated in the TOF analyzer. For the calculation of sum formulae, the monoisotopic mass averaged from at least three scans has been used. GC-MS data were obtained using an Agilent 6890N Network GC system including a 5973 mass selective detector and a 7683 injector system. GC and MS data were recorded using version SWG1701CA C.00.00 21-Dec-1999 of the Chemstation software by Agilent technologies. CD spectra were recorded on a Jasco 710 spectropolarimeter at room temperature in the range of 200–400 nm (step resolution 1 nm, speed 50 nm/min, response 2 sec, bandwidth 1 nm, sensitivity 10 mdeg, accumulation 3×). IR spectra were recorded on a Thermo Electron Corp. Nicolet IR 200 FT-IR (ATR mode, 64 scans). Column chromatography was carried out on Sephadex LH-20 (Amersham Biosciences, Uppsala, Sweden). Fractions were monitored by TLC (silica gel 60 254 nm) using toluene-ethyl acetate-acetic acid (7:3:0.1) as mobile phase (**1** black, **2** yellow after spraying with anisaldehyde sulphuric acid reagent). Analytical HPLC was performed on a DIONEX HPLC system using an ASI-100 autosampler and the UVD-340S diode array detector (200–595 nm). The system was running on a Gynkotek HP480 pump. An endcapped $RP_{18}$ column (125 × 4.6 mm, 5 $\mu$m) was used at a flow rate of 1.5 mL/min. A solvent system consisting of methanol (eluent A) and 0.1% (m/m) phosphoric acid (eluent B), starting from an initial ratio of 10% A and 90% B and reaching 100% A within 13.0 min, was used. Semi-preparative HPLC was performed on a YMC-Pack ODS-AQ column (250 × 20 mm, 10 $\mu$m) using a DIONEX HPLC system including a ASI-100 autosampler, a DIONEX P580 pump, a Gynkotek UVD-160 variable wavelength detector, and a Isco Foxy 200 fraction collector. All HPLC runs were recorded using the Chromeleon HPLC data system using version 6.30 SP1 built 587. All chemicals were used as received, solvents were distilled prior to use.

**Fungal Material.** Mature fruiting bodies of *G. pfeifferi* were collected on the grounds of Castle Ludwigsburg in the vicinity of Greifswald (Germany) in September 2002. They were taken from the same stump of *Fagus* sp. from which the fruiting bodies were collected for earlier studies of our group. They were kindly identified by Prof. Dr. H. Kreisel, Department of Biology, Ernst-Moritz-Arndt-Universität, Greifswald, Germany. A voucher specimen (herbarium accession number PI-28) is deposited at the Department of Pharmaceutical Biology, Ernst-Moritz-Arndt-Universität.

**Extraction and Isolation.** Air-dried and powdered fruiting bodies of *G. pfeifferi* (288 g) were extracted with $CH_2Cl_2$ in a soxhlet apparatus for 18 h to give a crude extract (8.5 g). The crude extract was rinsed with 450 mL of *n*-hexane-$CH_2Cl_2$ (2:7) on a Sephadex LH-20 column (open column, 3 × 35 cm, water cooled, flow 24 mL/h). The fraction not eluting was washed off with MeOH (F1-2, 2.9 g) and further fractionated into 100 fractions on a Sephadex LH-20 column (open column, 2 × 35 cm, water cooled, flow 20 mL/h) with 400 mL of MeOH-$H_2O$ (2:1). Every second fraction was analysed by HPLC as described above, and fractions containing **1** or **2** were combined and dried. Further purification by semi-preparative HPLC afforded 47 mg of compound **1** and 20 mg of compound **2**. Farnesylhydroquinone (**1**) was purified using a flow rate of 18 mL/min and a solvent system consisting of methanol (eluent A) and water (eluent B), starting from an initial ratio of 20% B for 2.5 min and then reaching 0% B within 8.5 min. For the isolation of ganomycin K (**2**), a gradient from 65% B reaching 45% B in 5 min, holding for 5 min and then reaching 0% B in 5 min with a flow rate of 16 mL/min was used.

**Synthesis of Farnesylhydroquinone (1). 1** has been synthesized as described in the literature.[19] Hydroquinone (437 mg) and farnesol (435 mg) were dissolved in 5 mL of anhydrous diethyl ether under argon. After addition of 0.4 mL of $BF_3$ etherate, the solution was stirred at room temperature for three hours. After addition of water, the product was extracted using diethyl ether and isolated using silica column chromatography (unoptimized yield: 23%).

**Ganomycin K (2).** (5*S*)-3-[(*E*)-7,8-dihydroxy-4,8-dimethylnon-3-enyl]-5-(2,5-dihydroxyphenyl)-furan-2(5*H*)-one; yellow oil; TLC toluene-EtOAc-$CH_3COOH$ (7:3:0.1) $R_f$ 0.26 (yellow after spraying with anisaldehyde-$H_2SO_4$ reagent); UV (MeOH) $\lambda_{max}$ (log ε) 203 (4.36), 298 (3.57) nm; CD (MeOH) $\Delta\varepsilon_{213}$ − 1.38 (π-π*), $\Delta\varepsilon_{233}$ + 7.13 (n-π*); IR $\nu_{max}$ 3282, 2935, 1736, 1645, 1585, 1504, 1454, 1375, 1280, 1200, 1159, 1080, 1054, 1024, 981, 814, 791, 740, and 714 $cm^{-1}$; $^1$H and $^{13}$C NMR (methanol-$d_4$, 600 MHz) see Table 1; negative ion HREIMS *m/z* 375.1820 (calcd for $C_{21}H_{27}O_6$ $[M - H]^-$, 375.1813, Δ 0.7 ppm). Complete NMR and MS raw data of ganomycin K (**2**) are available free of charge for download at http://dx.doi.org/10.6084/m9.figshare.686085.

**Antimicrobial Test System.** The antimicrobial tests were performed with Gram-positive bacteria (*S. aureus* ATCC 6538, *S. aureus* North German Epidemic Strain, *S. aureus* 34289, *S. aureus* 36881, *S. aureus* 38418, *S. aureus* 520, *S. aureus* 315,

*S. epidermidis* 847, *S. epidermidis* 125, *S. haemolyticus* 535) by the agar diffusion method. Ampicillin (SIGMA) and coenzyme Q10 (SIGMA) were used as controls. Substance-containing paper disks (6 mm diameter) were deposited on the surface of agar plates (nutrition agar, SIFIN, Berlin) seeded with overnight cultures of the test microorganisms. The plates were incubated for 18 h at 37 °C. After incubation the inhibition zones were measured and recorded as the diameter of the zone.

### Electronic Supplementary Material

Supplementary material is available in the online version of this article at http://dx.doi.org/10.1007/s13659-013-0036-5 and is accessible for authorized users.

### Acknowledgments

We thank K. Weisz for recording the NMR spectra. Financial support by the federal state of Mecklenburg-Vorpommern and the EU ("Landesforschungsschwerpunkt" EMAU 02 015 20) is gratefully acknowledged.

**Open Access** This article is distributed under the terms of the Creative Commons Attribution License which permits any use, distribution, and reproduction in any medium, provided the original author(s) and source are credited.

## Electronic supplementary material

Supplementary material, approximately 562 KB.
