# Supplementary material for: Isolation of farnesylhydroquinones from the basidiomycete Ganoderma pfeifferi
Source: Nat Prod Bioprospect. 2013 Jun 20;3(4):137–40. doi: 10.1007/s13659-013-0036-5 (PMC4131579; doi:10.1007/s13659-013-0036-5)

Isolation of Farnesyl Hydroquinones from the Basidiomycete *Ganoderma pfeifferi*

Timo H. J. Niedermeyer, Thomas Jira, Michael Lalk, Ulrike Lindequist

Spectra Farnesylhydroquinone (1) 2
- EI-MS Spectrum 2
- 1H NMR Spectrum 3
- 13C NMR Spectrum 5
- DEPT-135 NMR Spectrum 6
- COSY NMR Spectrum 7
- HSQC NMR Spectrum 8

Spectra Ganomycin K (2) 9
- EI-MS Spectrum 9
- UV and CD Spectra 9
- IR Spectrum 10
- 1H NMR Spectrum 101
- 13C NMR Spectrum 12
- COSY NMR Spectrum 13
- HSQC NMR Spectrum 14
- HMBC NMR Spectrum 15

Spectra Farnesylhydroquinone (1)

EI-MS Spectrum

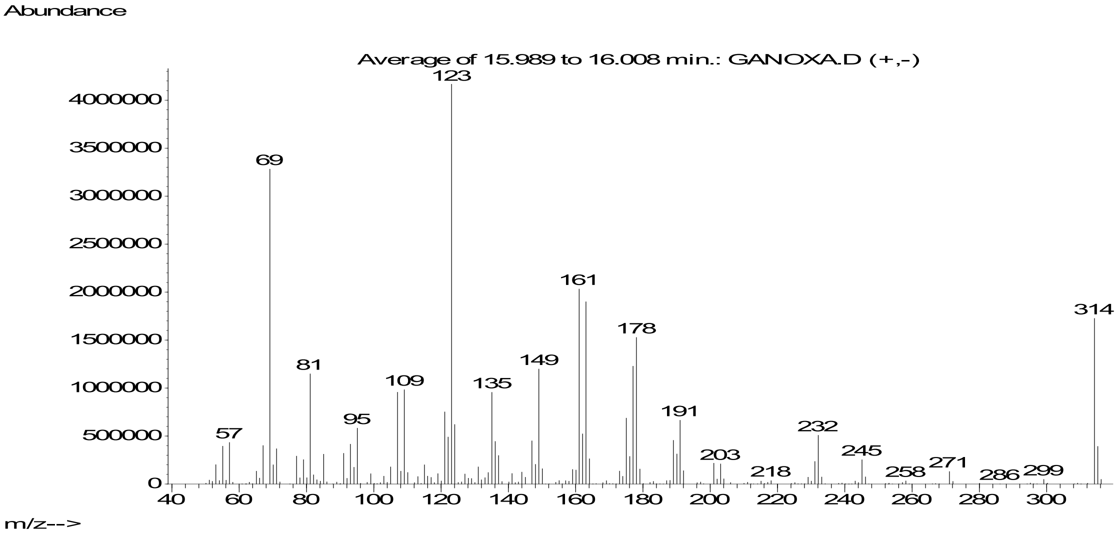

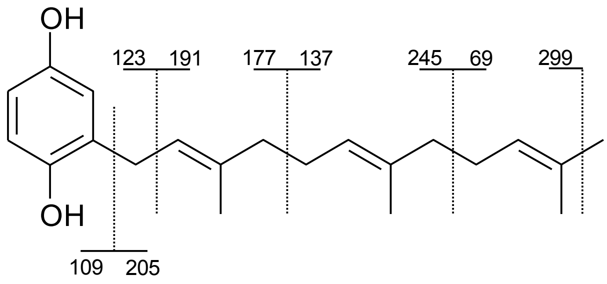

^{1}H NMR Spectrum

500 MHz in $CDCl_3$

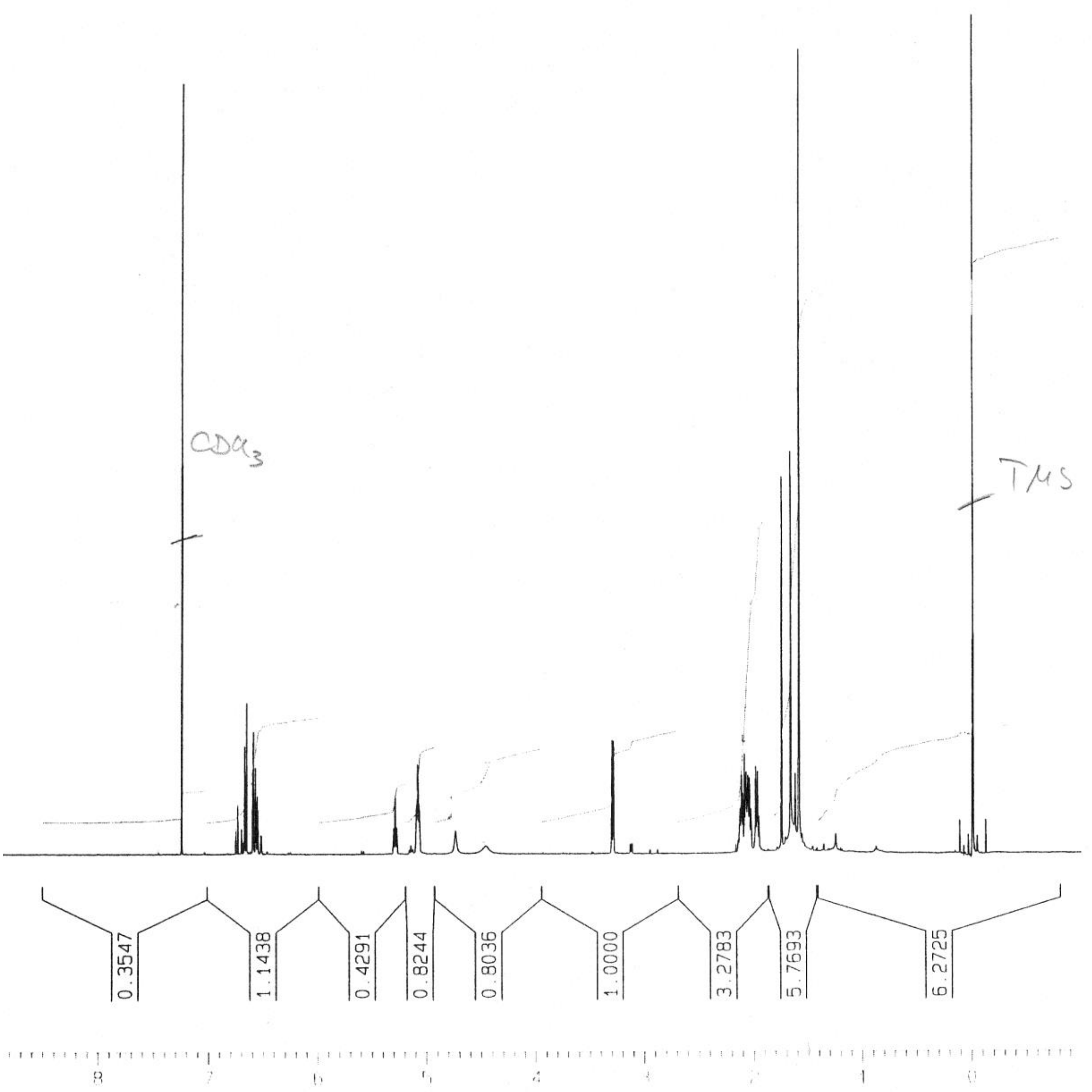

500 MHz in d_4-MeOH (with solvent suppression)

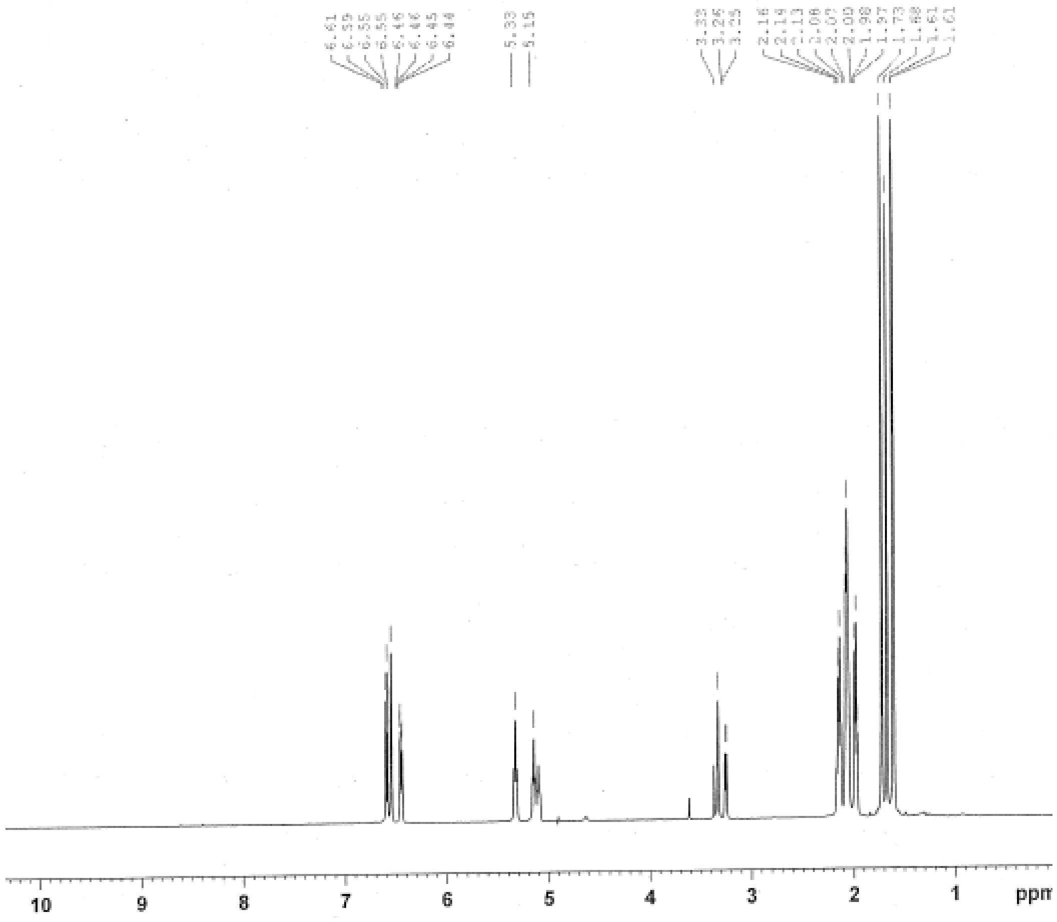

^{13}C NMR Spectrum

125 MHz in $CDCl_3$

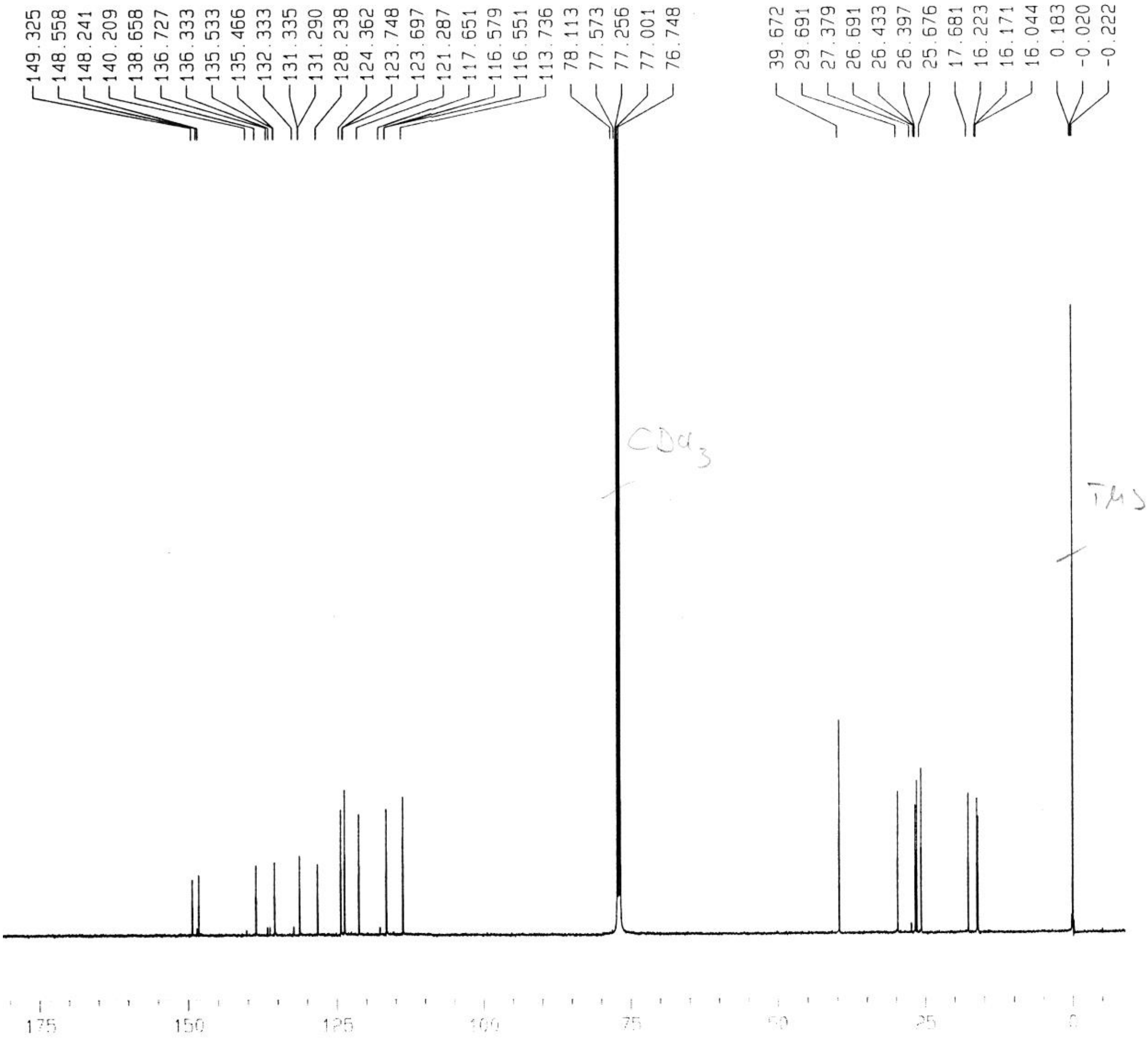

DEPT-135 NMR Spectrum

125 MHz in $CDCl_3$

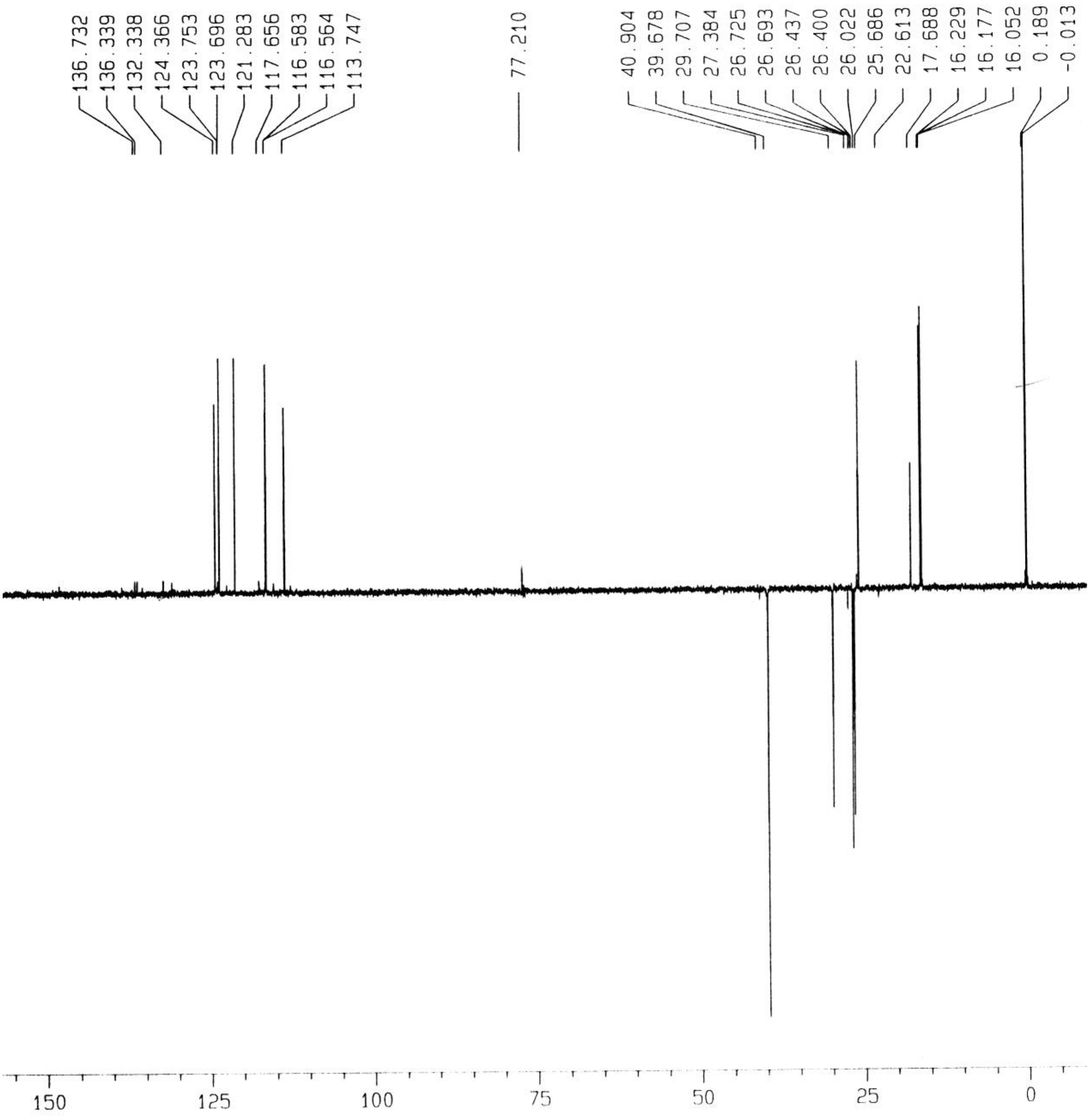

COSY NMR Spectrum

500 MHz in $CDCl_3$

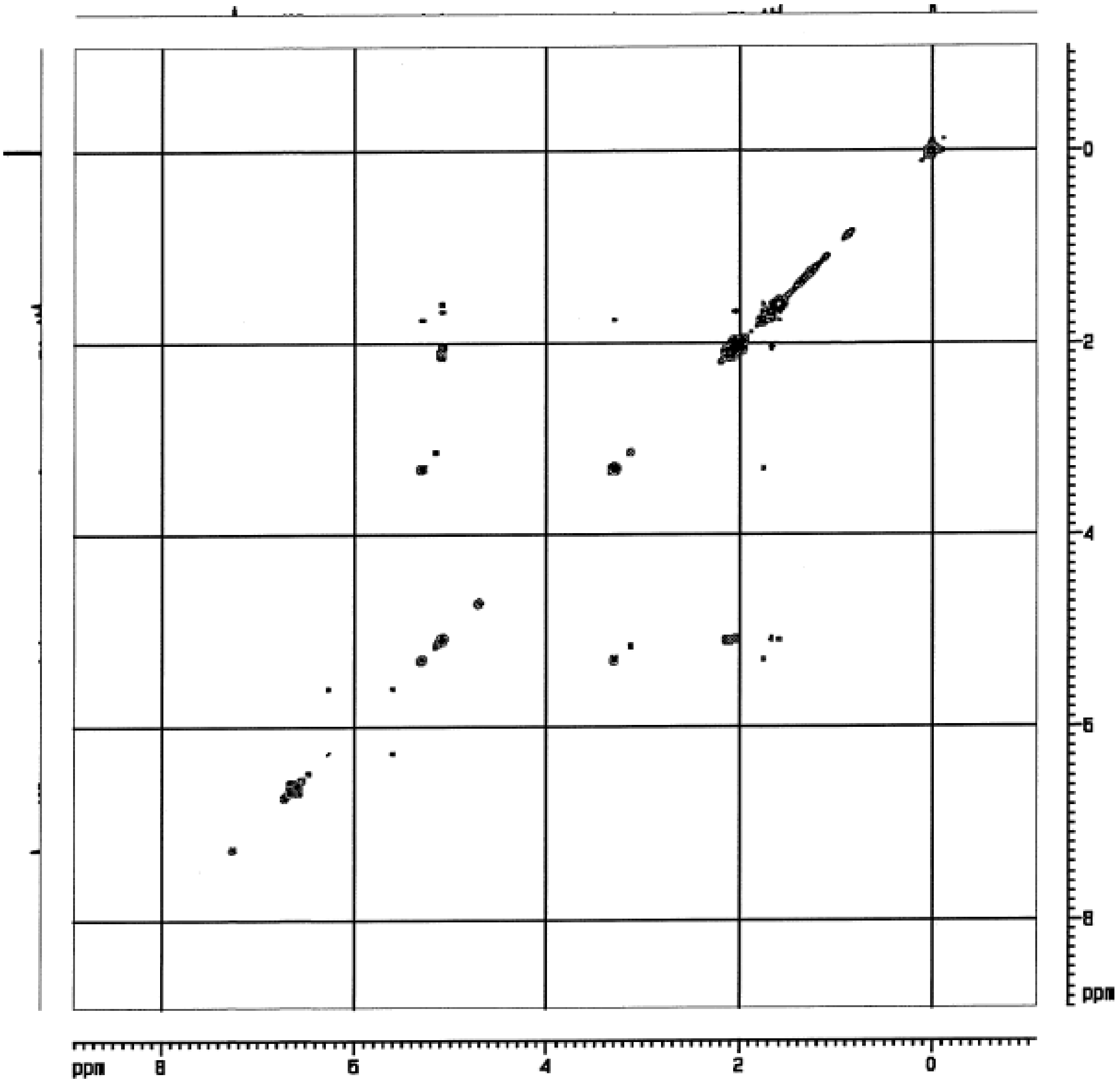

HSQC NMR Spectrum

500 / 125 MHz in d_4-MeOH

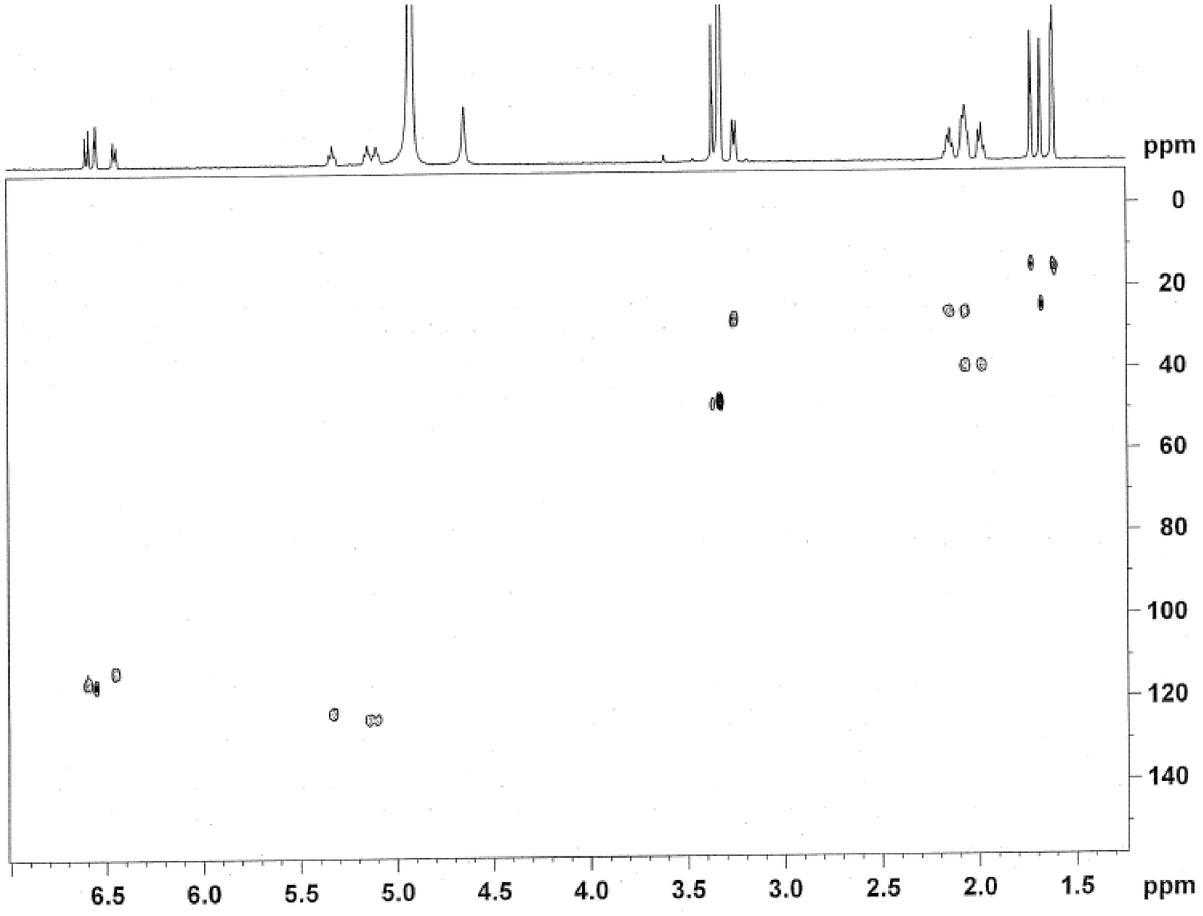

Spectra Ganomycin K (2)

EI-MS Spectrum

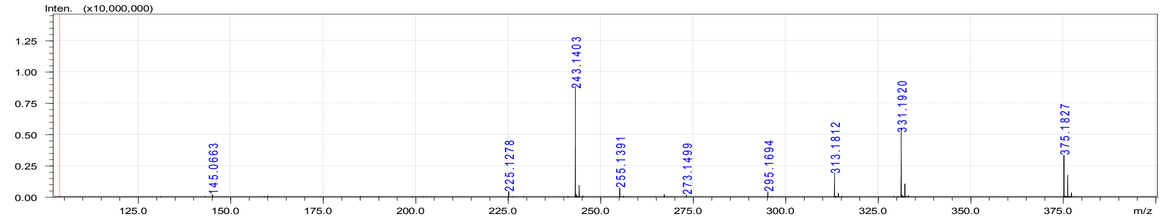

UV and CD Spectra

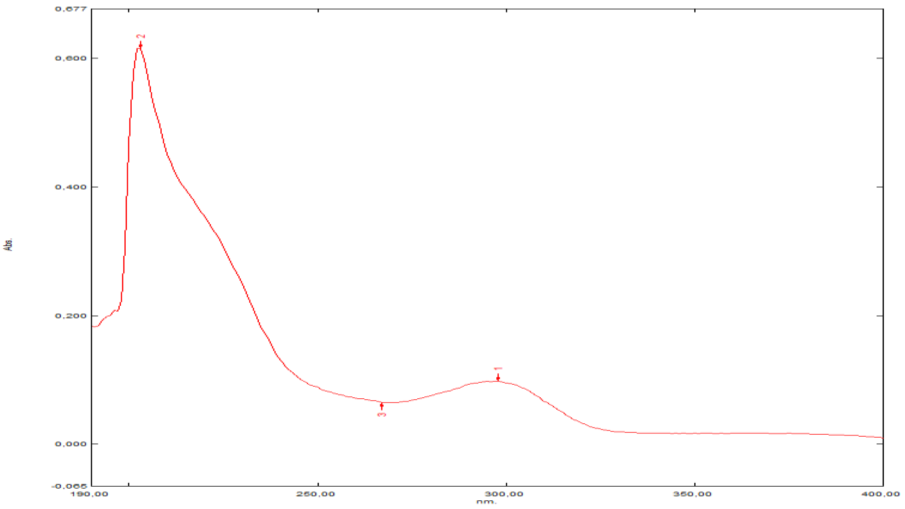

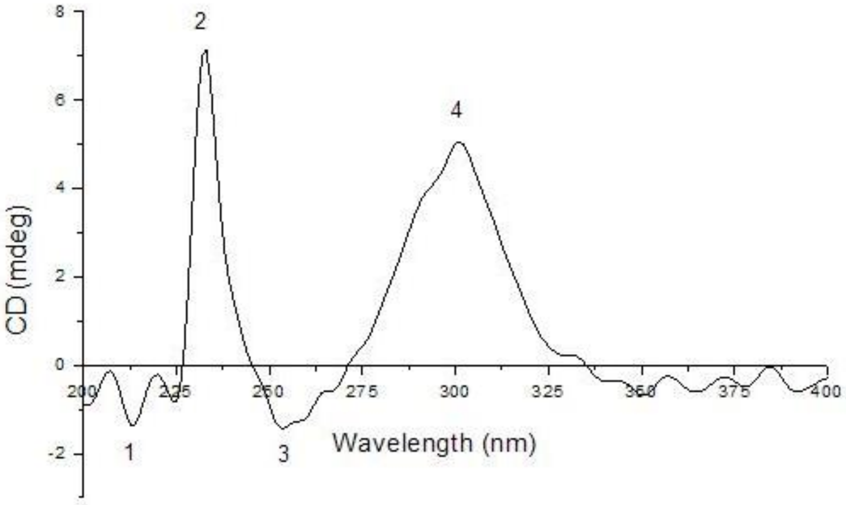

1:	213 nm	-1,38 mdeg
2:	233 nm	7,13 mdeg
3:	254 nm	-1,43 mdeg
4:	301 nm	5,06 mdeg

IR Spectrum

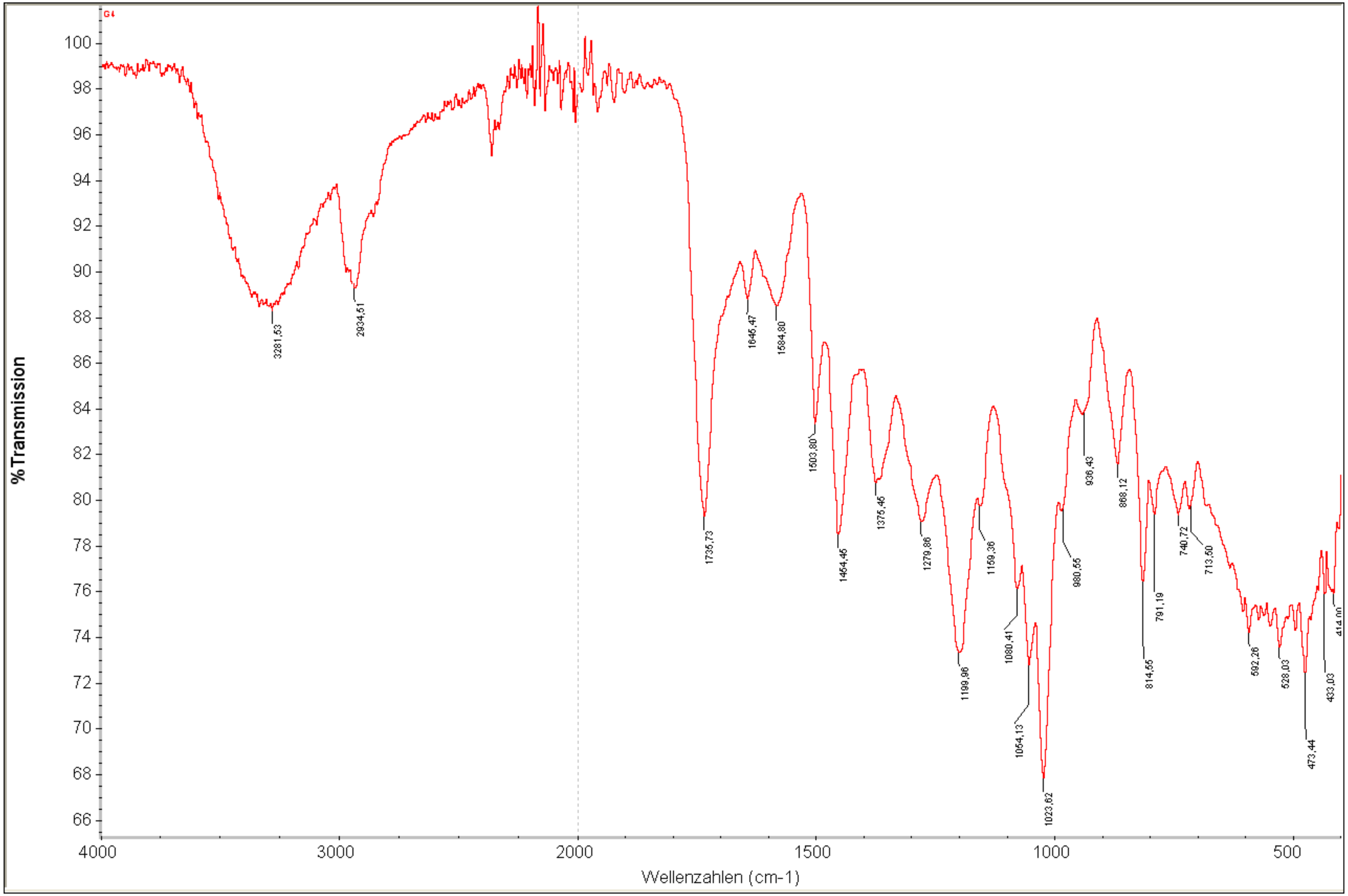

%Transmission
Wellenzahlen (cm-1)
3281,53
2934,51
1735,73
1645,47
1584,80
1503,80
1454,46
1375,46
1279,86
1199,96
1159,36
1080,41
1054,13
1023,62
980,55
936,43
868,12
814,55
791,19
740,72
713,60
592,26
528,03
473,44
433,03

^{1}H NMR Spectrum

600 MHz in d_4-MeOH

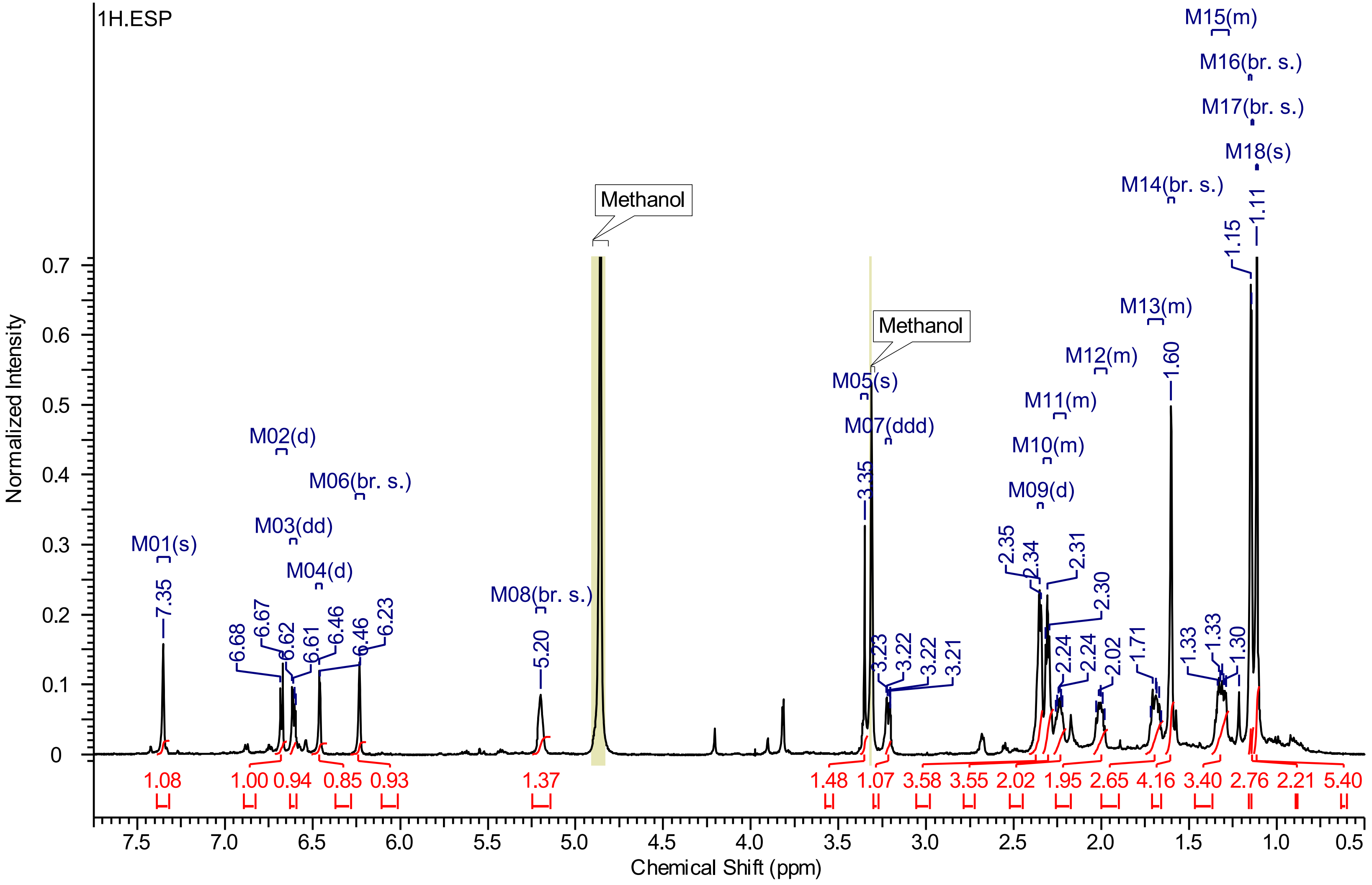

^{13}C NMR Spectrum

150 MHz in d_4-MeOH

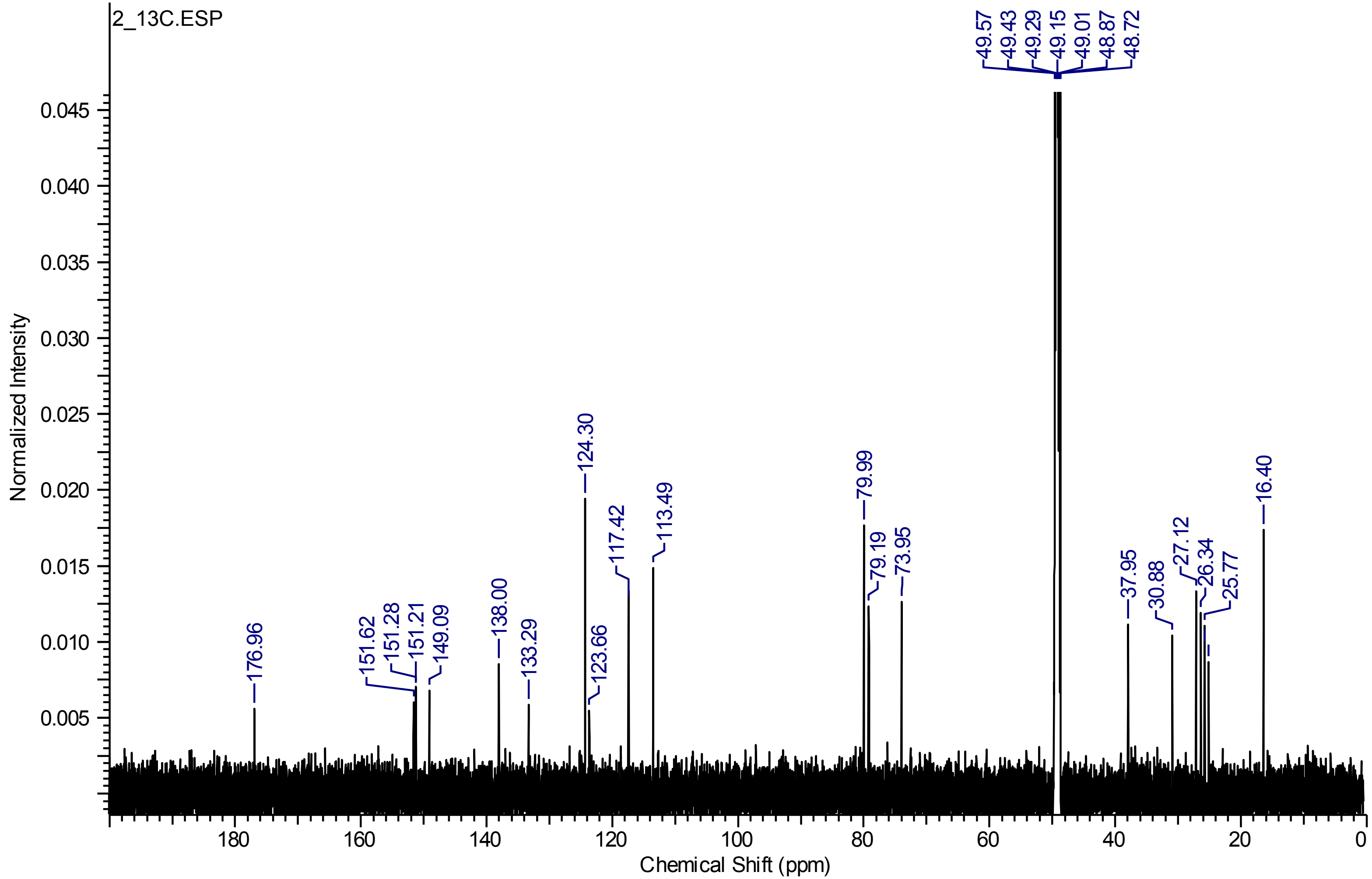

COSY NMR Spectrum

600 MHz in d_4-MeOH

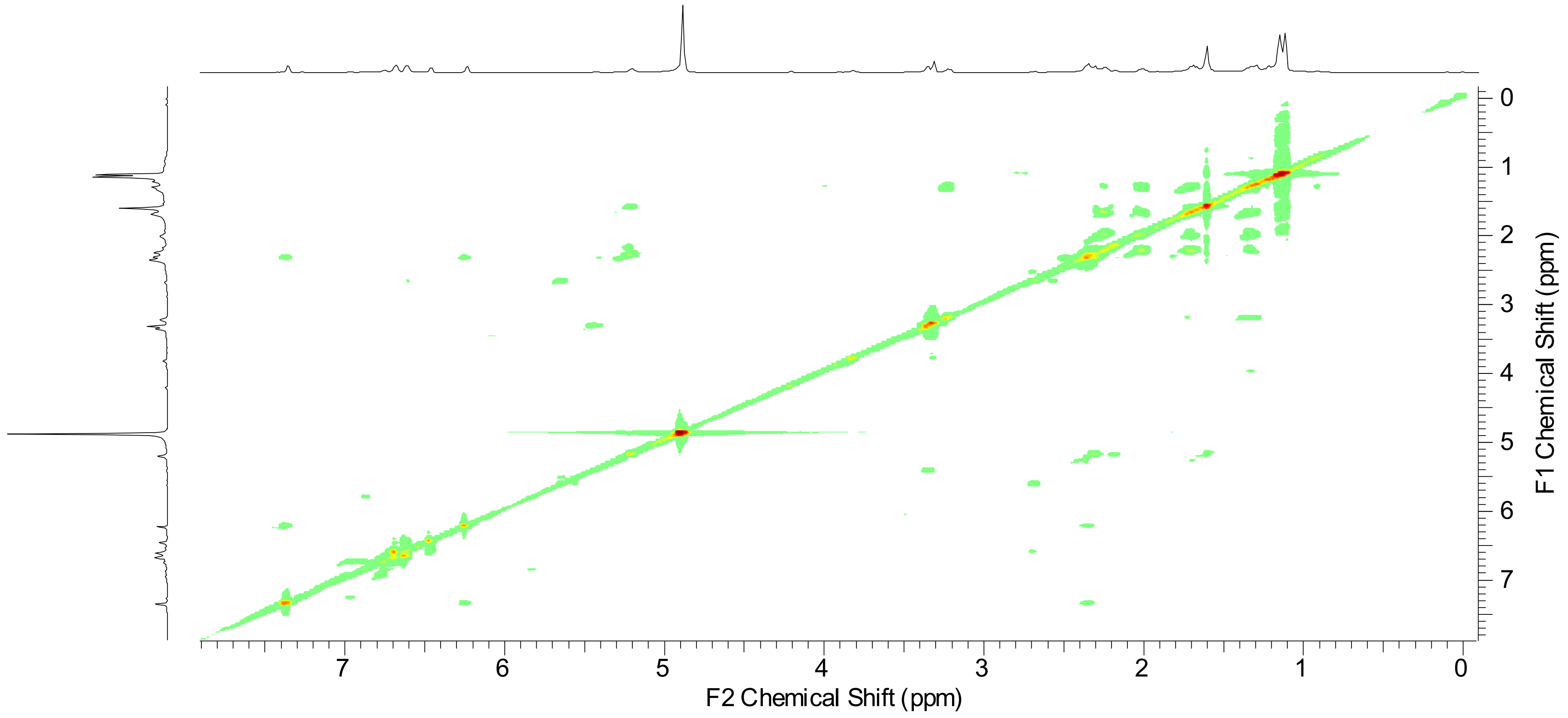

HSQC NMR Spectrum

600 / 150 MHz in d_4-MeOH

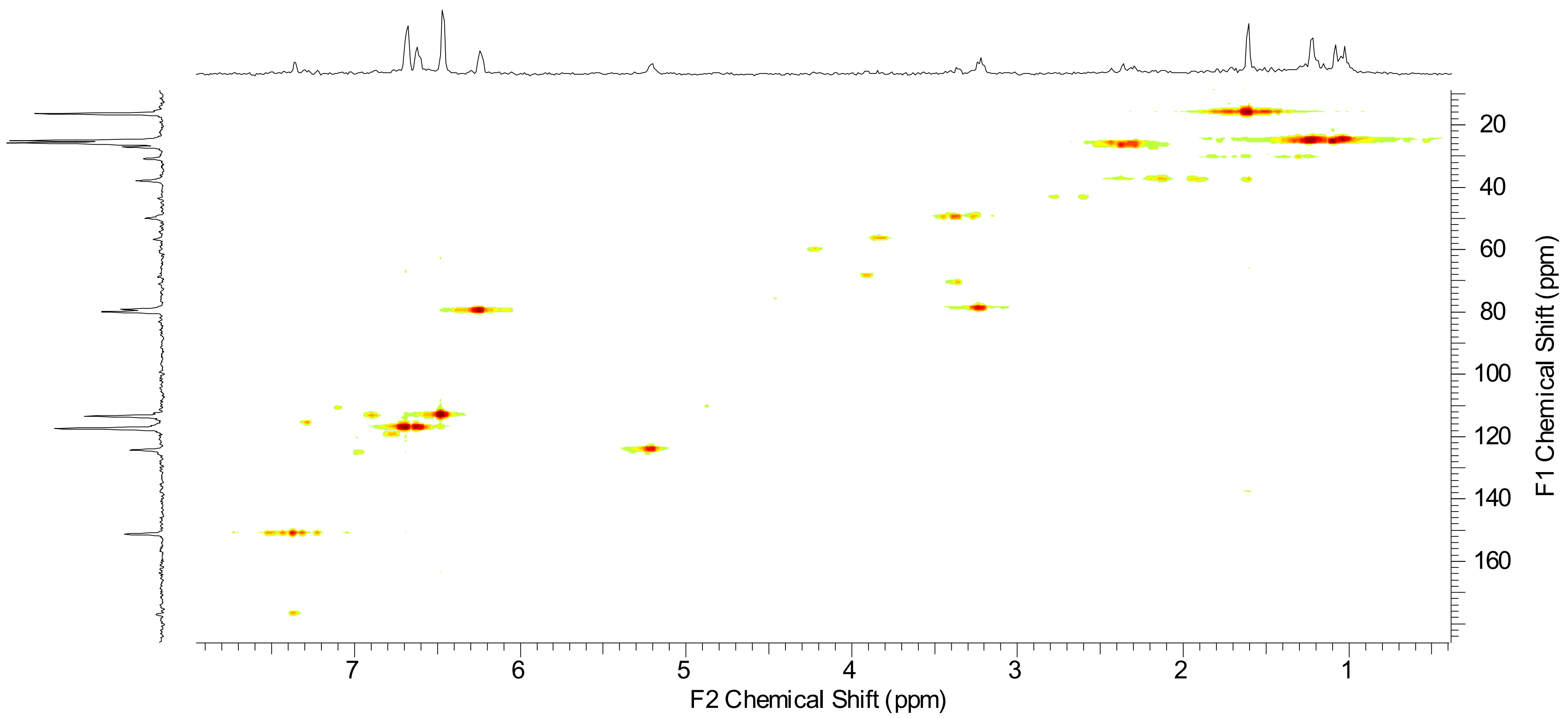

HMBC NMR Spectrum

600 / 150 MHz in d_4-MeOH

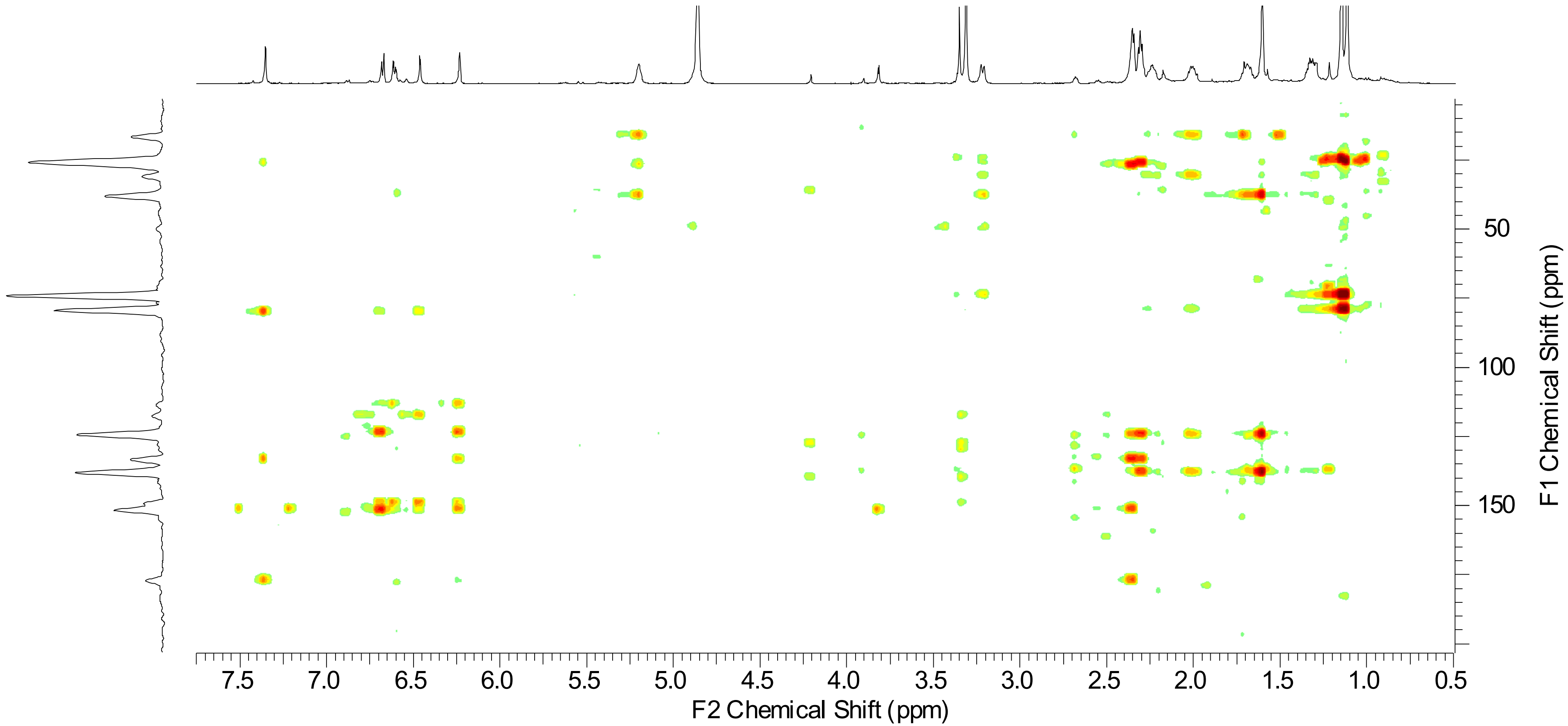

Supplement: Supplementary file 1 — Supplementary material, approximately 562 KB. [file 13659_2013_36_MOESM1_ESM.pdf]
